# Rare presentation of chronic ileocecal intussusception secondary to Burkitt’s lymphoma in three years Sudanese boy: a case report and literature review

**DOI:** 10.11604/pamj.2018.31.57.14793

**Published:** 2018-09-26

**Authors:** Atif Ahmed Mohamed Saad, Tasnim Kkalid, Mohammed Abbas, Karimeldin Mohammed Ali Salih

**Affiliations:** 1 Pediatrics Department, Faculty of Medicine, Om Dorman Islamic University, Sudan; 2 Pediatrics Department, Albulk Pediatric Teaching Hospital, MOH, Sudan; 3 Pediatrics Department, College of Medicine, Bisha University, KSA; 4 Pediatric Department, Faculty of Medicine, Kassala University, Sudan; 5 Pediatrics Department, Faculty of Medicine, University of Bahri, Sudan

**Keywords:** Intussusception, Burkett´s lymphoma, Sudan

## Abstract

A case report of chronic ileocecal intussusceptions in 3-years old Sudanese boy diagnosed as abdominal Burkett's lymphoma as leading point, who presented to his local hospital severely wasted with prolonged abdominal symptoms. Ultra sound and computed tomographic scan of his abdomen and pelvis with oral contrast confirmed intussusception. He was referred to pediatric surgical department and underwent laparotomy confirming ileocecal intussusception with resection of gangrenous part of his large and small bowel with end-to-end anastomosis. Histopathology of resected part showed infiltration of small bowel with cells of Burkett's lymphoma. This case highlights the importance of considering chronic intussusception, though rare, as a cause of faltering growth in young children with prolonged abdominal symptoms. The multidisplinary approach was highly appreciated and the outcome was satisfactory.

## Introduction

Intussusceptions, defined as insertion of proximal part of intestine into distal one, it is the second most common acute abdomen pathology after appendicitis [1]. Acute intussusception is one of the common causes of acute intestinal obstruction in children, but on seldom occasions associated with leading points to the intussusceptions. While chronic intussusceptions, which usually has history of more than 14 days, [2] is rare in childhood, commonly associated with leading points as neoplasm and other pathology [3]. It is rare to find the triad of acute intussusceptions, colicky intermittent abdominal pain, vomiting, and bloody stool in chronic intussusceptions [4]. Chronic intussusceptions as a clinical entity is poorly recognized and rarely included in the differential diagnosis of prolonged abdominal symptoms and faltering growth as reported in some studies [5]. It is frequently associated with a high rate of unsuccessful hydrostatic reductions. This makes an early surgical intervention advisable [6]. Chronic intussusception is a distinct clinical entity, characterized by intermittent attacks of abdominal pain lasting more than 14 days; other symptoms of acute intussusception may not present. One impressive clinical feature is significant weight loss due to long-standing anorexia and vomiting [5]. The incidence of chronic intussusception is about 3% of all cases of intussusception in children aged under one year and approximately 10% of children over that age [7]. Most pediatric ileocolic intussusceptions are idiopathic. In adults and occasionally in children over 2 years of age, a pathologic lead point for intussusception can be found [8]. In children, the incidence of identifiable lead point in pediatric intussusception has been reported as 1.5-12.0% [9]. Underlying pathological causes of intussusception can be identified in 1.5-12.0% of cases [9]. These include Meckel's diverticulum, polyps, duplications, mesentery cysts, intestinal hematoma and lymphoma [10] Lymphoma, although uncommon, arouses the most concern due to its malignant nature, and represents 6.5% of pathologic lead points of intussusception in children [9]. Navarro et al reported that long duration of symptoms and weight loss were two important clinical clues to the presence of gastrointestinal lymphoma [11] These symptoms were present in our case. Burkitt's lymphoma commonly presents as an abdominal mass and is often associated with abdominal pain, nausea and intestinal obstruction caused by direct compression of the bowel lumen or intussusception [12]. The peak age for gastrointestinal Burkitt's lymphoma in children is 5-15 years [13]. The distinct clinical entity of chronic intussusception is poorly recognized and rarely described [5] Rafinesque (1878) classified intussusception into 4 types: (a) hyper acute (dying within 2 days of onset), (b) acute (dying within a week), (c) subacute (surviving for 7-14 days) and (d) chronic (surviving for more than 14 days). Herewith, we describe a rare case of chronic intussusception secondary to Burkett's lymphoma in three years Sudanese boy: a brief review of the literature is presented.

## Patient and observation

A 3-years old male was admitted to his local hospital with history of vomiting associated with periods of constipation (for few days) followed by passage of formed stools or loose motions, those never contained blood. There was also colicky abdominal pain that referred to his penis with visible central bowel movements and abdominal distension that worsened with meals. He lost much of his weight although he was a healthy child before. He was exclusively breast fed only for the first six weeks of his life after which he depended mainly on boiled camel milk. Physical examination showed severely wasted boy with WHO z-scoring of his weight for length equal to-4SD, his length and head circumference on the 25^th^ centile for his age and sex. Abdominal examination revealed distended abdomen with visible peristalsis. Digital rectal examination revealed soft tissue mass 5 cm from the anal margin. He showed no response to nutritional supplementation. Complete blood count showed mild norm chromic anemia. Blood urea, serum keratinize and electrolytes were within the normal range. Abdominal and pelvic Ultra sound scan showed picture of intussusception with mesenteric lymph nodes enlargement. Computed tomography scan of his abdomen and pelvis with oral contrast showed large mass in the right iliac fossa indicating intussusception. Stool analysis showed hookworm infestation. He was screened for HIV, tuberculosis and celiac disease and all were excluded. Broad-spectrum antibiotics was started for suspected sepsis on admission. The patient was initially managed conservatively, given oral and injectable analgesics and nutritional supplementations with no or little response. Then referred to pediatric surgery after the confirmation of diagnosis and underwent laparotomy, eight cm of his terminal ileum, appendix and right hemi colon up to proximal part of his transverse colon was found to be gangrenous and was resected with end to end anastomosis. Histopathology of resected part showed infiltration of small bowel wall with starry sky patterned cells reaching serosal margins going with Burkitt's lymphoma. The boy did well post operatively, gain three-kilogram weight in the next four weeks. He was referred to the Radioisotopes center and subjected to further investigations for staging. Chemotherapy started with poor compliant was observed.

## Discussion

Chronic intussusceptions (CI) are mostly present with episodes of non-specific abdominal pain of prolonged duration of more than two weeks. Around 3% of all reported CI cases occur in infant less than one year, while about 10% in those over one year of age7 as in our case. A striking feature of chronic intussusception is emaciation and a marked loss of weight, which could be attributed to prolonged anorexia and vomiting and some reported cases have been misdiagnosed and investigated thoroughly for malabsorption including our case [3, 5]. Due to the high rate of unsuccessful hydrostatic reductions, all cases of CI should be treated surgically, as there is a high incidence of specific pathology, which needs surgical intervention. Moreover, some authors recommend other procedures, such as appendectomy or ileopexy, in addition to the manual reduction of the intussusception, in order to prevent recurrence [3]. Unfortunately, our patient had gangrenous bowel that could not be salvaged. The striking symptom of this boy's condition which was reported by his mum was the intermittent attacks of abdominal pain which referred to his penis. Referred penile pain, which was also described in other reported cases, was due either to the direct pulling effect of the mass on the kidney, or to the meso-colon dragging on the kidney, thus making it mobile and leading to traction effect on its nerves [14]. Chronic intussusception is rare but should be in the list of causes of faltering growth in young children especially those with prolonged vague abdominal symptoms. In this reported cases it was obviously evident that, this child has lymphoma, which is not spreaded to other area in addition to the fact that the child is doing fine without further chemotherapy to the best of our knowledge this was the first case to be reported in Sudan. In our culture people have strong believe in camel milk for treatment for many conditions.

## Conclusion

This case highlights the importance of considering chronic intussusception, though rare, as a cause of faltering growth in young children with prolonged abdominal symptoms. The multidisplinary approach was highly appreciated and the outcome was satisfactory.

## Competing interests

The authors declare no competing interests.
